# UPLC-ESI-QTRAP-MS/MS Analysis to Quantify Bioactive Compounds in Fennel (*Foeniculum vulgare* Mill.) Waste with Potential Anti-Inflammatory Activity

**DOI:** 10.3390/metabo12080701

**Published:** 2022-07-27

**Authors:** Maria Assunta Crescenzi, Gilda D’Urso, Sonia Piacente, Paola Montoro

**Affiliations:** 1 Department of Pharmacy, University of the Study of Salerno, Via Giovanni Paolo II 132, I-84084 Fisciano, Italy; mcrescenzi@unisa.it (M.A.C.); piacente@unisa.it (S.P.); 2 Ph.D. Program in Drug Discovery & Development, Department of Pharmacy, University of the Study of Salerno, Via Giovanni Paolo II 132, I-84084 Fisciano, Italy

**Keywords:** vegetable waste, metabolomic analysis, anti-inflammatory activity

## Abstract

*Foeniculum vulgare* is a perennial aromatic plant whose cultivation produces large amounts of waste rich in bioactive compounds with promising anti-inflammatory activities. Nine selected metabolites were quantified through Ultra Performance Liquid Chromatography (UPLC) hyphenated to QTRAP mass spectrometry by using MRM (multiple reaction monitoring) was performed on four parts of fennel: bulb, stem, little stem, and leaf. Analysis revealed significant differences in the amount of quantified metabolites, suggesting that little stem and leaf are the most valuable parts of the waste. Phenolic acids and glycosylated flavonoids were quantified for their known possible anti-inflammatory activities; in fact, due to this reason their ability to inhibit COX-1 and COX-2 isoforms was evaluated through a fluorometric assay, resulting in specific inhibitors of COX-2 at certain concentrations. In conclusion, as the leaf of fennel may be beneficial to human health, clinical studies should include it in nutraceuticals or functional foods for human consumption.

## 1. Introduction

Inflammation is a pathophysiological process that allows the body to defend itself against various pathogens such as viruses and microbes. The inflammatory response alters blood flow, enhancing the permeability of blood vessels and the migration of fluids, proteins, and white blood cells from the circulation to the site of tissue damage [1]. An inflammatory response that lasts only a few days is called acute inflammation, while a longer-lasting response is called chronic inflammation, which involves a high amount of proinflammatory cytokines and is correlated with several serious disorders such as arthritis, asthma, colitis, and neurodegenerative diseases including Alzheimer’s and Parkinson’s diseases [2]. Anti-inflammatory drugs are categorized into two classes: steroidal (SAIDs) and nonsteroidal anti-inflammatory agents (NSAIDs). Steroidal drugs inhibit the cellular processes that lead to the synthesis of proinflammatory and immunostimulant substances and activate cellular processes that lead to the synthesis of anti-inflammatory and immunosuppressive substances. Nonsteroidal anti-inflammatory drugs act by blocking reversibly the binding site of cyclooxygenases 1 and 2 (COX-1 and COX-2) [3]. Both therapies have side effects, although NSAIDs to a lesser extent [4].

For this reason, plant-originated compounds, with potential anti-inflammatory activity, might be useful in treating many diseases, especially in chronic inflammation, thereby reducing the drug dosage and its side effects [5,6]. Flavonoids are one of the majority classes among plant constituents [7]. Some flavonoids exert their anti-inflammatory activity mostly by regulating the expression of proinflammatory elements such as pro-inflammatory enzymes such as COX-2 and inducible nitric oxide synthase (iNOS), and by arresting the “cytokine storm”, inhibiting proinflammatory cytokines such IL-1 and IL-6 [8,9,10].

*Foeniculum vulgare* (Apiaceae/Umbelliferae), well-known as fennel, is an aromatic plant, which was initially cultivated in Asia Minor and Mediterranean regions [11]. Previous work confirmed the chemical characterization of fennel, in particular of the waste of *F. vulgare*, underlining a high content of polyphenolic compounds, especially flavonoids [12]. The large amount of fennel by-products obtained from the agri-food compartment can be useful to produce nutraceuticals, nutrients in foods with beneficial effects on health [13]. 

This work aims to define which part of fennel waste is richest in bioactive compounds and therefore the part useful for the preparation of extracts and nutraceuticals with the most promising biological activities, including the anti-inflammatory one, to be addressed mainly to the management of chronic inflammation. In the present work, four different parts of fennel waste were analyzed: bulb, stem, little stem, and leaf. Nine selected metabolites were quantified using UPLC hyphenated to QTRAP mass spectrometry by using MRM (multiple reaction monitoring) mode. This analysis revealed large differences in the content of quantified metabolites, identifying the little stem and the leaf as the parts of fennel waste richest in bioactive compounds. 

Our previous work highlighted the antioxidant activity of *F. vulgare* waste [12], so the knowledge of the biological activities of this plant matrix was deepened. In fact, the potential anti-inflammatory activity of the major compounds identified in *F. vulgare* was evaluated through a fluorometric COX inhibitory assay, determining the ability of these metabolites to inhibit COX-1 and COX-2 enzymes. Finally, the major presence of the most active compounds in each fennel part was underlined, with the results showing the leaf as the most promising part among the fennel waste.

## 2. Results and Discussion

### 2.1. UPLC-ESI-QTRAP-MS/MS Quantitative Analysis

In order to evaluate the different concentrations of the majority compounds expressed in the various parts of *F. vulgare*, quantitative UPLC-ESI-QTRAP-MS/MS analysis was carried out. Standard optimization was performed to improve the spectral parameters of each compound, presented in Table 1 with the precursor/product transitions. 

**Figure 1:**
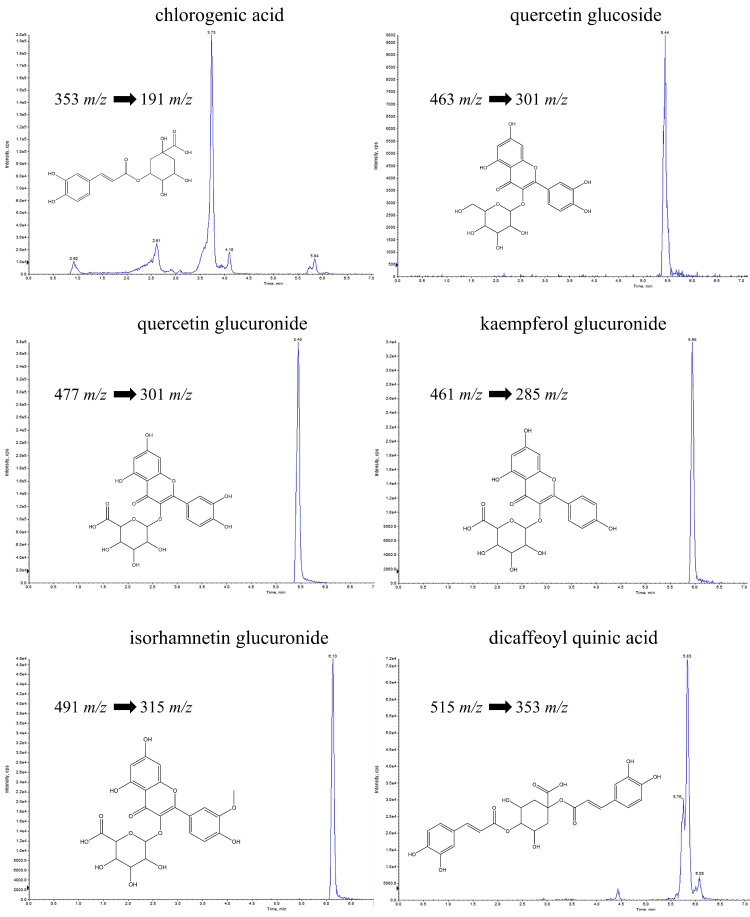
Q1/Q3 mass transitions for chlorogenic acid, quercetin glucoside, quercetin glucuronide, kaempferol glucuronide, isorhamnetin glucuronide, and dicaffeoylquinic acid obtained by UHPLC-ESI-QTRAP-MS/MS analysis and selected for MRM analysis of leaf extract (FVLE).

Preliminarily ESI-QTRAP-MS/MS spectra were analyzed by direct infusion of standards into the ESI source of a mass spectrometry instrument equipped with a triple quadruple analyzer. The transitions of the ESI/MS/MS analysis were recorded to set a selective and sensitive UPLC-ESI-QTRAP-MS/MS method through MRM mode (Figure 1).

**Figure 2:**
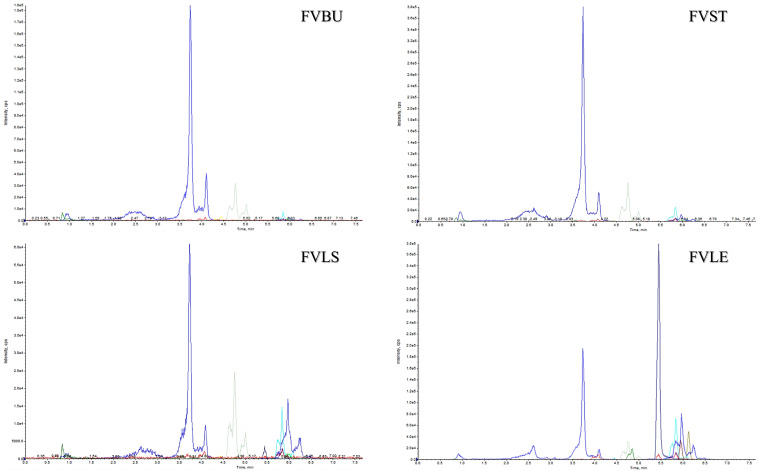
UHPLC-ESI-QTRAP-MS/MS profiles of bulb (FVBU), stem (FVST), little stem (FVLS), and leaf (FVLE) extracts of *F. vulgare* through analysis in MRM mode.

From the results of the quantitative analysis, it is clear that the metabolite profiles of the little stem and the leaf are more similar than those of the bulb and the stem (Figure 2), according to previous work [12]. Indeed, the bulb and the stem are richer in phenolic acids, presenting quantities of 25.39 and 58.70 mg of neochlorogenic acid (1) and 65.25 and 152.32 mg of feruloyl quinic acid (4), respectively. There is evidence of the anti-inflammatory activity of phenolic acids; they can decrease the amount of arachidonic acid and its metabolites. They are able to inhibit COX enzymes, so reducing the eicosanoids, prostaglandins, and thromboxane in the inflammatory processes [14].

The greatest amount of dicaffeoylquinic acid (2) was found in the stem and the leaf (27.82 and 41.17 mg), as its derivate, dicaffeoylquinic acid malonyl (9), which was identified in each sample, reaching the highest concentration in the leaf. It is clear even from a qualitative analysis of UHPLC-ESI-QTRAP-MS/MS profiles (Figure 2) that the little stem and the leaf are the parts with the highest amount of bioactive compounds. Particularly, the leaf is the only part of *F. vulgare* containing glucuronate flavonoids. This research highlighted above all a high quantity in fennel leaves of quercetin glucuronide (5) and isorhamnetin glucuronide (6). As a result of glucuronidation, physiological properties of flavonoids are significantly altered, including their solubility, bioactivity, bioavailability that usually increases, intercellular transport, and excretion. Importantly, the position of glucuronidation is also related to antioxidant and pro-oxidant properties of flavonoids; for instance, glucosides and 3-*O*-glucuronides of resveratrol have higher antioxidant capabilities than trans-resveratrol [15].

### 2.2. Method Validation

As claimed by EMA guidelines, the UPLC-ESI-QTRAP-MS/MS method was proved [16]. Calibration curves were achieved by plotting the area of external standard beside the noted concentration of each metabolite; each standard solution was examined in triplicate. The linearity was good for each metabolite in the concentration range of analysis (correlation coefficients from 0.997 to 0.999).

In order to determine the limit of detection (LOD) and the limit of quantification (LOQ), each standard compound was serially diluted, under optimized conditions, to achieve signal-to-noise ratios (S/N) of 3:1 and 10:1. Accordingly, the LOD ranged from 0.002–0.010 mg/L and the LOQ ranged from 0.002–0.08 mg/L, indicating good sensitivity of the developed method.

Three of any given sample were analyzed on the same day and three more aliquots over three consecutive days, one aliquot for each day. The precision of the method was calculated by expressing it as a percentage relative standard deviation (RSD). 

We evaluated extraction efficiency by performing recovery experiments with the optimized parameters. UPLC-ESi-QTRAP-MS/MS experiments were performed using three different concentration levels (high, middle, and low) in triplicate at each concentration level for three standard solutions. Within the same day, there was a good recovery and precision, with recovery rates ranging from 95% to 105%.

### 2.3. Anti-Inflammatory Activity

Preliminary in vitro experiments were set up to evaluate the hypothesis that some of the bioactive compounds identified in *F. vulgare* may exhibit potential anti-inflammatory activity by inhibiting the two isoforms of cyclooxygenase, the constitutive COX-1 and the inducible COX-2. The anti-inflammatory activity potential of the classes of compounds most expressed in fennel waste is confirmed in the literature [17,18,19,20]. Their antioxidant activity also provides a basis for evaluating their potential anti-inflammatory activity [21]. In fact, inflammation and oxidative stress are usually associated [22]. The increase of free radicals such as superoxide is usually associated with inflammation in chronic diseases. Atherosclerosis, cataracts, and inflammation may be caused by the imbalance of these chemical species [23].

A fluorometric assay, therefore, was conducted to evaluate the inhibition of this enzyme by quercetin glucuronide, kaempferol glucuronide, isorhamnetin glucuronide, quercetin glucoside, and dicaffeoylquinic acid, the metabolites contained more in the various studied extracts, evaluating the reduction of resorufin fluorescence, a product of the reaction between PGG_2_ and ADHP (10-acetyl-3,7-duhydroxyphenoxazine) by the peroxidase component of COXs.

Results of the assay are reported in Table 2, expressed as IC_50_ (µM). All metabolites were found to have biological activity. In the table, the values of IC_50_ of SC-560 and DuP-697 (positive controls, selective for COX-1 and COX-2) were reported, determined by using the same analysis conditions for COX-1 and COX-2 assays, respectively.

In general, the main metabolites in fennel showed a higher IC_50_ for COX-1 than for COX-2. Specifically, metabolites with a more promising anti-inflammatory activity capable of inhibiting the inducible isoform COX-2 were quercetin glucoside, dicaffeoylquinic acid, and isorhamnetin glucuronide, with an IC_50_ corresponding to 9.34, 14.77 and 15.88 µM, respectively.

## 3. Experimental Design

### 3.1. Raw Materials

**Figure 3:**
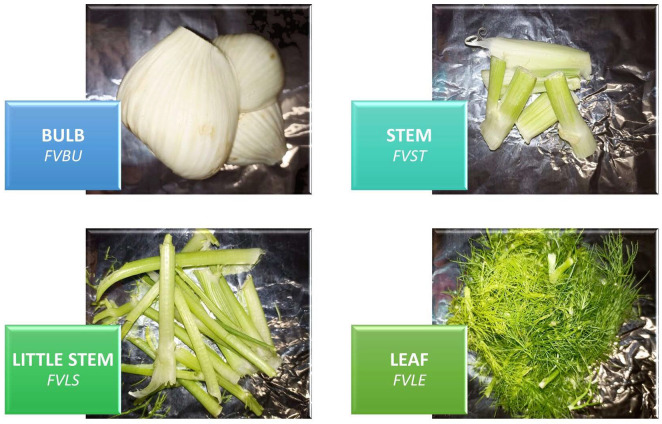
*F. vulgare* parts analyzed: FVBU (*F. vulgare* bulb), FVST (*F. vulgare* stem), FVLS (*F. vulgare* little stem) and FVLE (*F. vulgare* leaf).

Waste of *F. vulgare* was supplied by Paolillo (Eboli, Salerno, Italy), a company specializing in the production and marketing of fennel. The by-products were retrieved from fennel cultivation of the variety Tiziano cultivated in the locality of Campomarino in Puglia and collected in December 2019. The bulb, the superficial leaves, and both large and small stems of fennel were supplied as parts of the plant. Based on this, the samples were classified into the following four groups: FVBU—*F. vulgare* bulb, FVST—*F. vulgare* stem, FVLS—*F. vulgare* little stem, FVLE—*F. vulgare* leaf (Figure 3).

### 3.2. Chemicals

Ethanol and water used for the extractions were bought from VWR (Milan, Italy). Acetonitrile (ACN), formic acid, water, and methanol of LC-MS grade were purchased from Romil. The anti-inflammatory kit was bought from Cayman Chemicals, cat. Number 700100 (Ann Abor, MI, USA). The standards used for the quantification, neochlorogenic acid, dicaffeoylquinic acid, quercetin glucoside, feruloyl quinic acid, quercetin glucuronide, isorhamnetin glucuronide, and kaempferol glucuronide, were purchased from Sigma-Aldrich (Milan, Italy).

### 3.3. Sample Preparation

The different parts of the *F. vulgare* waste were divided and stored at −80 °C and then freeze-dried. The freeze-dried plant materials were extracted with a sonication with a solution of ethanol/water (80:20) previously described [12].

An ultrasonic bath was used to extract one gram of dried drugs from the FVBU and FVST samples with 20 mL of ethanol/water (80:20) for 15 min. For the FVLS and FVLE samples, a volume of 40 mL was required per gram of matrix. Filter paper was used to filter the extracts after three rounds of extraction. LC-MS analysis was performed on each sample by preparing a solution of methanol with a final concentration of 1 mg/mL of the extract, which was dried using a stream of nitrogen.

### 3.4. Quantitative Analysis

#### 3.4.1. ESI-QTRAP-MS and ESI-QTRAP-MS/MS Analyses

Full scan ESI-QTRAP-MS and collision induced dissociation (CID) ESI-QTRAP-MS/MS analyses of standards were performed on an ABSciex (Foster City, CA, USA) API6500 QTRAP spectrometer. 

In order to optimize the analytical parameters, a standard solution of each metabolite (1 µg/mL in methanol) was infused into the source at a flow rate of 10 L/min. Data were acquired in the negative ion MS and MS/MS mode.

#### 3.4.2. UPLC–ESI-QTRAP-MS Analyses in MRM (Multiple Reaction Monitoring) Modality

Quantitative analysis of the bioactive compounds of *F. vulgare* waste was carried out with a Shimadzu Nexera LC system in line with a Sciex 6500 QTRAP MS equipped with an Omega C18 column (Phenomenex, Aschaffenburg, Germany) (100 × 2.1 mm i.d., 1.6 μm). The mobile phases used were water +0.1% formic acid (A) and acetonitrile +0.1% formic acid (B). The metabolites were chromatographically separated using the following increasing linear gradient (*v*/*v*) at a flow rate of 0.300 mL/min of solvent B: 0.1–2.13 min, from 5 to 15%; 2.13–6.40 min, from 15 to 35%; 6.40–8.53, from 35 to 80% and then back to 5% for 1.53 min. The ion mode was negative and 5 μL of each sample was used for injection. The 6500 QTRAP was set up for IonSpray operation, and the compounds were detected using multiple reaction monitoring (MRM). Mass spectrometry source parameters were set up as follows: curtain gas (CUR) = 35; collision gas (CAD) = medium; ion spray voltage (IS) = −4500; temperature (TEM) = 350; ion source gas 1 (GS1) = 25; ion source gas 2 (GS2) = 25. The transitions of each analyzed metabolite are reported in Table 1 with the parameters (declustering potential (DP), entrance potential (EP), collision energy (CE), collision cell exit potential (CXP)) determined with the infusion of the standard solution to have the maximal response. The dwell time for each analyte was 20 ms. There were typically 14 points across all chromatographic peaks with a total cycle time of 0.3 s. Analyst software 1.6.2 was used for data acquisition and processing (ABSciex, Foster City, CA, USA).

#### 3.4.3. Method Validation

UPLC-ESI-QTRAP-MS/MS method was validated according to the European Medicines Agency guidelines (EMA quality guidelines ICH Q2) to validate analytical methods; in particular precision, specificity, linearity, limit of quantification (LOQ) and limit of detection (LOD). Precision was determined by analyzing five different concentrations for each metabolite with triplicate intra-day assays and inter-day assays over 3 days. The specificity of a technique is measured by its inability to be interfered with by other analytes detected in the area of interest. Linearity was identified by correlation values of calibration curves. In order to determine the limit of quantification (LOQ; equivalent to sensitivity), we injected a series of progressively diluted standard solutions until a signal-to-noise ratio of 10 was achieved. Various dilutions of standard solutions were injected until the signal-to-noise ratio reached three to estimate the limit of detection (LOD).

### 3.5. Anti-Inflammatory Activity

Phytochemicals such as polyphenolic acids are plant-based polyphenols with diverse biological functions, working as ancestors of bioactive molecules that are regularly used in the pharmaceutical, cosmetic, and food industries [24]. Indeed, there is a lot of evidence on the biological activities of these compounds, such as anti-inflammatory, inhibiting, for example, NF-kB or the expression of anti-inflammatory mediators [25].

Anti-inflammatory activity was evaluated determining the capacity of some major metabolites of *F. vulgare* to inhibit the cyclooxygenase activity (COX). The COX-1 and COX-2 inhibitory assay was carried out using a COX Inhibitor Screening Assay Kit (Catalogue N 700100, Cayman Chemicals, Ann Abor, MI, USA) following the instructions provided by the manufacturer. The assay uses the peroxidase component of COXs. The reaction between PGG_2_ and ADHP creates resorufin, a highly fluorescent compound. Its fluorescence was evaluated with an excitation wavelength of 535 nm and an emission wavelength of 590 nm. The inhibition of COXs is directly proportional to the reduction of resorufin fluorescence. The anti-inflammatory activity was evaluated using metabolites of fennel waste in different concentrations (25, 50, 100, and 150 µM) and the results are expressed as inhibitory activity (IC50, µM), according to the following Equation (1):

% Inhibition = ([Initial Activity − Sample Activity])/(Initial Activity) × 100.(1)

Potent synthetic inhibitors selective for COX-1 and COX-2 presence in the kit were used as a positive controls in different concentrations: SC-560 (2, 4, 8 and 10 nM) and DuP-697 (10, 20, 40 and 80 nM).

The analyses were made in triplicate and the results are expressed as the mean of the IC_50_ (µM) ± the standard deviation.

## 4. Conclusions

This research has confirmed and deepened the role of *F. vulgare* waste as a resource of bioactive compounds with promising anti-inflammatory activity [12]. Therefore, recovering and reusing these wastes can benefit human health.

UPLC-ESI-QTRAP-MS/MS quantitative analysis has demonstrated that the bulb and the stem are a source of phenolic acids such as neochlorogenic acid and ferulic acid.

The fennel waste richest in bioactive compounds is the leaf. Additionally, phenolic acids are also rich in glycosylated and glucuronate flavonoids. 

Researchers in the past decades have become interested in them for their antioxidant, anti-inflammatory, and antibacterial properties [26].

According to the COX inhibitory fluorometric assay, kaempferol, isorhamnetin, and quercetin glucuronide may have anti-inflammatory properties. These compounds have shown IC_50_ of COX-2 lower than those exhibited for COX-1. These results are important because the compounds may be capable of inhibiting, at specific concentrations, the isoform COX-2 that is activated by inflammation.

COX inhibitors that also inhibit isoform COX-1 have gastric side effects since COX-1 is responsible for the protective and homeostatic action of prostaglandins [27]. 

In conclusion, this study demonstrated that *F. vulgare* waste contains several bioactive compounds, opening up the way to in vivo studies on cells to evaluate how these metabolites are really involved in the inflammatory phenomenon.

There may be a potential benefit to human health from consuming the leaf and, therefore, it would be interesting to create functional foods containing it to address chronic inflammation. 

## Figures and Tables

**Table 1:** Quantitative data obtained from the analysis of selected phenolic compounds in *F. vulgare* extracts using UHPLC–ESI-QTRAP-MS/MS analyses in MRM mode.

Compounds		DP	EP	CE	CXP	PI	DI	FVBU	FVST	FVLS	FVLE
1	neochlorogenic acid	−60	−4	−24	−17	353	191	25.39 ± 1.44	58.70 ± 0.69	6.80 ± 0.06	17.10 ± 0.86
2	dicaffeoylquinic acid	−61	−4	−24	−38	515	353	6.59 ± 0.09	27.82 ± 0.17	11.15 ± 0.21	41.17 ± 0.00
3	quercetin glucoside	−138	−9	−28	−32	463	301	nd	nd	3.16 ± 0.67	50.85 ± 0.00
4	feruloyl quinic acid	−89	−4	−33	−21	367	191	65.25 ± 0.00	152.32 ± 6.86	42.47 ± 0.00	46.77 ± 1.20
5	quercetin glucuronide	−52	−8	−35	−30	477	301	nd	nd	1.14 ± 0.11	163.76 ± 2.61
6	isorhamnetin glucuronide	−52	−8	−35	−30	491	315	0.67 ± 0.07	0.97 ± 0.38	0.89 ± 0.13	257.90 ± 4.23
7	kaempferol glucuronide	−59	−8	−35	−30	461	285	nd	nd	0.14 ± 0.00	37.84 ± 1.28
8	coumaroyl quinic acid *	−60	−4	−24	−17	337	191	6.42 ± 0.23	1.58 ± 0.05	nd	nd
9	dicaffeoylquinic acid malonyl *	−61	−4	−24	−38	601	395	4.44 ± 0.24	11.73 ± 1.68	19.98 ± 1.01	48.18 ± 4.65

Mean in mg/100 g dried weight with the standard deviation, DP declustering potential, EP entrance potential, CE collision energy, CXP collision cell exit potential, PI product ion, DI daughter ion. FVBU (*Foeniculum vulgare* bulb), FVST (*Foeniculum vulgare* stem), FVLS (*Foeniculum vulgare* little stem), FVLE (*Foeniculum vulgare* leaf). * Compounds quantified without standards (nd: not detected).

**Table 2:** Anti-inflammatory potential evaluation of polyphenolic compounds of *F. vulgare* measured as inhibition of COX-1 and COX-2 enzymes, expressed as the media of three determinations of the IC_50_ (µM) ± SD (standard deviations). SC-560 and DuP-697 are selective inhibitors of COX-1 and COX-2, respectively.

Compounds	IC50 (µM)	
	COX-1	COX-2
Kaempferol glucuronide	228.38 ± 16.81	47.93 ± 3.50
Isorhamnetin glucuronide	94.72 ± 1.22	15.88 ± 1.16
Quercetin glucoside	103.46 ± 8.66	9.34 ± 1.11
Dicaffeoylquinic acid	85.57 ± 10.94	14.77 ± 2.39
Quercetin glucuronide	570.83 ± 40.00	26.28 ± 1.55
SC-560 (COX-1)	0.005 ± 0.001	/
DuP-697 (COX-2)	/	0.025 ± 0.001

## Data Availability

The data presented in this study is contained within the article.
